# Identification and Characterization of Wall-Associated Kinase (WAK) and WAK-like (WAKL) Gene Family in *Juglans regia* and Its Wild Related Species *Juglans mandshurica*

**DOI:** 10.3390/genes13010134

**Published:** 2022-01-12

**Authors:** Mengdi Li, Jiayu Ma, Hengzhao Liu, Mengwei Ou, Hang Ye, Peng Zhao

**Affiliations:** Key Laboratory of Resource Biology and Biotechnology in Western China, Ministry of Education, College of Life Sciences, Northwest University, Xi’an 710069, China; mengdili@nwu.edu.cn (M.L.); majiayu@stumail.nwu.edu.cn (J.M.); hengzhaoliu@stumail.nwu.edu.cn (H.L.); oumengwei@stumail.nwu.edu.cn (M.O.); sxsdyehang@hotmail.com (H.Y.)

**Keywords:** *Juglans*, tandem duplication, wall-associated kinase gene family

## Abstract

Wall-associated kinase (WAK) and WAK-like kinase (WAKL) are receptor-like kinases (RLKs), which play important roles in signal transduction between the cell wall and the cytoplasm in plants. *WAK/WAKLs* have been studied in many plants, but were rarely studied in the important economic walnut tree. In this study, 27 and 14 *WAK/WAKL* genes were identified in *Juglans regia* and its wild related species *Juglans mandshurica*, respectively. We found tandem duplication might play a critical role in the expansion of *WAK/WAKL* gene family in *J. regia*, and most of the *WAK/WAKL* homologous pairs underwent purified selection during evolution. All WAK/WAKL proteins have the extracellular WAK domain and the cytoplasmic protein kinase domain, and the latter was more conserved than the former. *Cis*-acting elements analysis showed that *WAK/WAKL* might be involved in plant growth and development, plant response to abiotic stress and hormones. Gene expression pattern analysis further indicated that most *WAK/WAKL* genes in *J. regia* might play a role in the development of leaves and be involved in plant response to biotic stress. Our study provides a new perspective for the evolutionary analysis of gene families in tree species and also provides potential candidate genes for studying *WAK/WAKL* gene function in walnuts.

## 1. Introduction

The cell wall is the first barrier against the invasion of pathogens and also plays a key role in the maintenance of cell structure in plants [1,2]. When exposed to external stimuli, the cell wall transmits signals into the cell via carriers on it. Protein kinase is a key signal carrier and plays a critical role in plant growth and development. Receptor-like protein kinases (RLKs) are one kind of important protein kinases that participate in cell signal transduction as the receptors of various signal molecules [3]. Studies showed that RLKs generally consist of three crucial domains, including the extracellular ligand-binding domain, the hydrophobic transmembrane domain and the cytoplasmic serine/threonine (Ser/Thr) kinase catalytic domain [4,5]. As a relatively unique RLKs, wall-associated kinases (WAKs) have epidermal growth factor (EGF) outside of the cell, which are directly and closely connected to the cell wall and play irreplaceable roles in plant cell signal transduction [6,7]. WAK-like (WAKL) proteins were similar to WAK proteins in the structure, both of them have the extracellular WAK and Ser/Thr kinase domains, while the EGF domain was specific to WAK proteins [7]. WAK/WAKLs are the only kind of receptors involved in cell wall signaling, which can directly transfer signals from extracellular to cytoplasm, and they also work in plant cell expansion, metal tolerance, plant disease resistance and response to various plant hormones and abiotic stresses [8,9,10,11].

*WAK/WAKL* genes have been identified in Arabidopsis, rice and cotton [7,12,13]. *AtWAK1* has been proven to play a role in the aluminum tolerance of roots in Arabidopsis [9]. The expression of *AtWAKL4* is induced by several ions (such as Na+, K+ and Zn2+), and the tolerance of plants to ions is related to its expression level [14]. In addition, the decrease of *AtWAK4* expression resulted in shorter primary roots, accompanied by impaired lateral root and inhibited root hair elongation in Arabidopsis [15]. Another study showed that the decrease in AtWAK2 protein content makes cells lose their ability to expand, suggesting that WAK proteins could participate in the plant cell expansion process [16]. It was found that the transcription of *OsWAK1* was significantly induced by *Magnaporthe oryzae* infection in rice, and the expression accumulation of *OsWAK1* in sensitive varieties was much higher than that in resistant varieties, suggesting that WAK proteins plays an important role in the pathogen defense of plants [17]. A recent study found that *GhWAK7A* is involved in regulating the responses to the Fusarium and Verticillium wilts in cotton [10]. Moreover, another study showed that *GhWAKL* also participate in improving the resistance to Verticillium wilt in cotton [18]. Similar studies have been reported in other plant species. For example, *TaWAK7D* plays a positive role in the defense response of wheat to *Rhizoctonia cerealis* infection [11]. ZmWAK-RLK1 can improve the resistance of corn to leaf blight by reducing the infiltration of pathogens into host tissues [19]. *WAKL*, encoded by *Stb6*, improves the pathogen resistance of wheat without causing a hypersensitivity reaction, and proves that *WAKL* was involved in plant disease resistance [20]. Therefore, the *WAK/WAKL* gene family plays a significant role in the resistance to biotic and abiotic stresses in plants.

Common walnut (*Juglans regia*) and its wild related species *Juglans mandshurica* are both economic forest species with significant economic, nutritional and medicinal value [21]. In recent years, the whole genomes of these two *Juglans* species have been sequenced, which has laid a foundation for our comparative study of them [22,23]. It is of great significance to study the characteristic differences of key gene families associated with stress response in these two *Juglans* species. Our previous study found that there were four gene families associated with disease resistance (including the *WAK* gene family) that evolved rapidly in *J. manshurica*, compared with *J. regia* [23]. *WAK/WAKL* genes has been found in many plants, such as Arabidopsis [12], rice [13], cotton [3,7], rose [24] and barley [25], suggesting that they may be essential to the development and growth of plants. However, we know little about the *WAK/WAKL* gene family in the commercially valuable tree species *J. regia* and *J. mandshurica*. Therefore, it is necessary and meaningful to identify this gene family in two *Juglans* species and conduct a comprehensive comparative study between them. Here, we identified and analyzed the *WAK/WAKL* gene family in *J. regia* and its wild related species *J. mandshurica* for the first time. Specifically, we performed phylogenetic relationship, conserved domain, chromosome localization, gene duplication event and gene structure analysis in WAK/WAKL members of *J. regia* and *J. mandshurica*. We also used multi-organ and biotic stress transcriptome data to generate the expression profiles of all identified *WAK/WAKL* genes, as well as analyzed *cis*-acting elements analysis in gene promoter regions, providing vital clues for the investigation of *WAK/WAKL* gene function in two walnut species (*J. regia* and *J. mandshurica*). In addition, our study provides a reference for further study of genetic changes in walnut (*Juglans*) species after evolution and domestication.

## 2. Materials and Methods

### 2.1. Identification of WAK/WAKL Members in J. regia and Its Wild Related Species J. mandshurica

To obtain candidate members of the *WAK/WAKL* gene family in two *Juglans* species, the protein sequences of AtWAK/WAKL (https://www.arabidopsis.org/, accessed on 12 December 2021) were used as query sequences and all protein sequences of *J. regia* (https://www.ncbi.nlm.nih.gov/genome/, accessed on 12 December 2021) *J. mandshurica* [23] and *Quercus robur* [26] were used as databases for Blastp with E-value < 1E-5. The protein domains of all candidate members were then analyzed to determine the final WAK/WAKL members, as detailed below. Firstly, candidate members that did not contain WAK or GUB_WAK_bind domains and STKc_IPAK or PKc_like super family domains were removed by searching the CDD database in NCBI (https://www.ncbi.nlm.nih.gov/cdd/?term=, accessed on 12 December 2021). Secondly, all final WAK/WAKL members in *J. regia*, *J. mandshurica* and *Q. robur* were obtained by eliminating members without WAK or GUB_WAK_bind and Pkinase_Tyr or Pkinase domains using Pfam database (http://pfam.xfam.org/, accessed on 12 December 2021). Finally, the members that contain the EGF or EGF_CA domain (SMART database; http://smart.embl.de/, accessed on 12 December 2021), signal peptide (SignalP-5.0; https://services.healthtech.dtu.dk/service.php?SignalP-5.0, accessed on 12 December 2021) and transmembrane domain (TMHMM-2.0; https://services.healthtech.dtu.dk/service.php?TMHMM-2.0, accessed on 12 December 2021) were identified as WAK members, and the rest were WAKL members [7].

### 2.2. Chromosome Localization and Gene Duplication Mode Analysis

The location of *WAK/WAKL* genes on chromosomes was mapped using TBtools software [27]. The identification of gene duplication modes and collinearity analysis was accomplished by MCScanX software [28] using the genome file of two *Juglans* species. The TBtools software was also used to calculate the Ka/Ks ratio [27].

### 2.3. Physicochemical Properties and Subcellular Localization Prediction

The physicochemical properties of all identified WAK/WAKL proteins were predicted from online websites (http://www.expasy.org/tools/protparam.html, accessed on 12 December 2021). Subcellular localizations of all identified members were predicted using an online site WoLF PSORT (https://wolfpsort.hgc.jp/, accessed on 12 December 2021). 

### 2.4. Phylogenetic Tree Construction and Gene Structure Analysis

Phylogenetic trees were constructed by TBtools software [27] using protein sequences from Arabidopsis, *Gossypium hirsutum*, *Q. robur*, *J. regia* and *J. mandshurica* (model: JTT+F+R7; method: Maximum Likelihood; bootstrap: 1000). Gene structure analysis was performed using the online website GSDS (http://gsds.gao-lab.org/, accessed on 12 December 2021). ClustalX was used for multi-sequence alignment and Weblogo was used for sequence logo display (http://weblogo.threeplusone.com/, accessed on 12 December 2021). The phylogenetic tree was embellished using the online site iTOL (https://itol.embl.de/, accessed on 12 December 2021).

### 2.5. Cis-Acting Element Prediction and Gene Expression Analysis

*Cis*-acting element prediction was performed using 2000 bp sequences upstream of the identified *WAK/WAKL* genes at PlantCARE (http://bioinformatics.psb.ugent.be/webtools/plantcare/html/, accessed on 12 December 2021). The multi-organ gene expression data were derived from our previous transcriptome sequencing data. The sequencing data about pathogen stress on walnuts were downloaded from the published public Sequence Read Archive database (https://www.ncbi.nlm.nih.gov/geo/query/acc.cgi?acc=GSE147083, accessed on 12 December 2021; accession number GSE147083) [29]. The raw data were filtered by fastp [30], and the clean reads were mapped to the reference genome using HISAT2 [31]. Then, the gene expression was calculated using FeatureCounts [32]. Heatmaps were drawn using TBtools software [27].

## 3. Results

### 3.1. Genome-Wide Identification and Phylogenetic Analysis of the WAK/WAKL Gene Family in J. regia and Its Wild Related Species J. mandshurica

The whole genome information of *J. regia* [22] and its wild related species *J. mandshurica* [23] was used to identify the WAK/WAKL members in them. AtWAK/AtWAKL proteins from Arabidopsis [12] were used as the query sequences to screen out candidate WAK/WAKL proteins in two selected *Juglans* species. Through the domain screening of candidate proteins, 11 WAK and 16 WAKL protein encoding genes were identified in *J. regia*, and 5 WAK and 9 WAKL protein encoding genes were identified in *J. mandshurica*. All identified WAK/WAKL proteins contain the WAK and protein kinase domain, and only the WAK protein contain the EGF domain in this study. Moreover, all identified WAK proteins have a transmembrane domain and signal peptide domain. To facilitate the follow-up study, we renamed all members according to the order of their location on the chromosomes. The gene IDs, gene names and protein sequences of all identified WAK/WAKL members are shown in Table S1.

**Figure 1:**
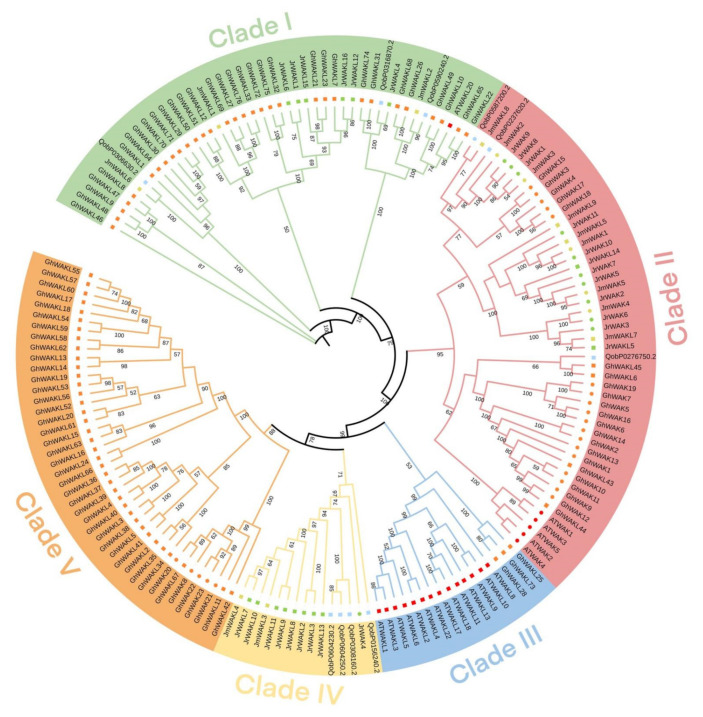
The Maximum Likelihood phylogenetic tree (ML) of WAK/WAKL proteins of *Arabidopsis thaliana*, *Gossypium hirsutum*, *Quercus robur*, *Juglans regia* and *Juglans mandshurica*. Circles and squares represented WAKs and WAKLs, respectively. Red, orange, blue, green and yellow represented WAK/WAKLs of *A. thaliana*, *G. hisutum*, *Q. robur*, *J. regia* and *J. mandshurica*, respectively.

A Maximum Likelihood (ML) phylogenetic tree was constructed using WAK/WAKL protein sequences of Arabidopsis, *G. hirsutum*, *Q. robur*, *J. regia* and *J. mandshurica* (Figure 1), and we then studied and classified their evolutionary relationships based on the tree. All members of the WAK/WAKL family were grouped into five clades (Clade I-V). The largest clade was Clade II, which contains 10 JrWAKs, 2 JrWAKLs, 5 JmWAKs, 4 JmWAKLs, 18 GhWAKs, 4 GhWAKLs, 1 QrWAK, 2 QrWAKLs and 5 AtWAKs. Clade IV contains only WAK/WAKL members of woody plants (*Q. robur*, *J. regia* and *J. mandshurica*), and Clade V contains only GhWAKLs. Most WAK and WAKL proteins clustered separately in the phylogenetic tree, indicating that the difference between WAK and WAKL protein sequences was greater than that between species. In Clades II-V, the WAK/WAKLs of woody plants showed a clustered distribution, while that of Arabidopsis and cotton showed the clustered distribution phenomenon, suggesting that the WAK/WAKLs of the three woody plants had a relatively close genetic relationship. The majority of WAK/WAKL proteins from Arabidopsis were clustered separately (with just one exception, AtWAKL20), suggesting that WAK/WAKL members might have evolved independently after the speciation of two *Juglans* species and Arabidopsis.

### 3.2. Physicochemical Property and Subcellular Localization Analysis of the WAK/WAKL Proteins in J. regia and Its Wild Related Species J. mandshurica

The length of WAK/WAKL proteins in *J. regia* ranged from 513 amino acids (aa, JrWAKL9) to 839 aa (JrWAK2), with an average length of 716 aa (Table 1). In contrast, WAK/WAKL proteins in *J. mandshurica* were longer in length, ranging from 629 aa (JmWAKL1) to 1396 aa (JmWAK3), with an average length of 832 aa. The molecular weight (MWs) of the JrWAK/WAKL proteins ranged from 57.57 kDa (JrWAKL8) to 92.13 kDa (JrWAK4), with an average of 79.61 kDa. Similarly, the MWs of JmWAK/WAKL proteins was heavier than that of JrWAK/WAKL, ranging from 69.19 kDa (JmWAKL2) to 155.95 kDa (JmWAK3), with an average of 92.99 kDa. In addition, 19 and 10 WAK/WAKL proteins were acidic protein (isoelectric point < 7) in *J. regia* and *J. mandshurica*, respectively. There were only 6 and 5 WAK/WAKL proteins with instability index values greater than 40 in *J. regia* and *J. mandshurica*, respectively. Almost all of the identified WAK/WAKL proteins had a negative grand average of hydropathicity (GRAVY) values (with only one exception) in the selected two species, indicating that these WAK/WAKL proteins are hydrophilic. As expected, most identified WAK/WAKL was located in the plasma membrane (Table 1).

### 3.3. Protein Domain and Gene Structure Analysis of the WAK/WAKL Members in J. regia and Its Wild Related Species J. mandshurica

**Figure 2:**
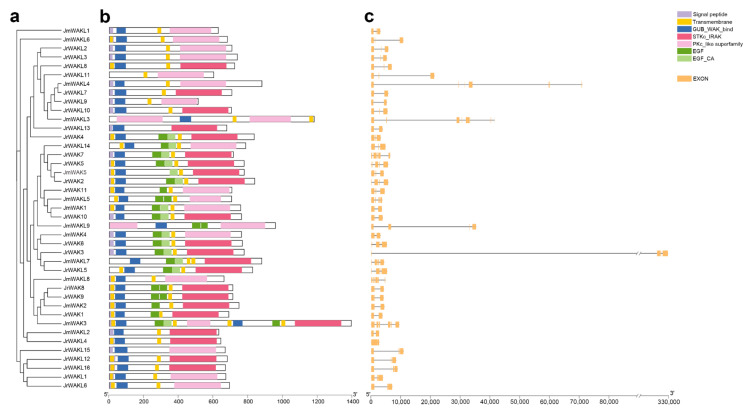
The gene structures and protein domains of WAK/WAKL members. (**a**) Maximum likelihood phylogenetic tree of WAK/WAKLs in two Juglans species; (**b**) Protein domains of WAK/WAKLs in two *Juglans* species. Different domains were represented by different colored boxes; (**c**) Gene structures of *WAK/WAKLs* in two *Juglans* species. Yellow boxes indicated exons, and gray lines indicate introns.

To show the protein domain and gene structure of WAK/WAKLs in terms of similarity, we first constructed a phylogenetic tree using their protein sequences (Figure 2a). WAK/WAKL proteins were found to contain highly conserved domains. Specifically, both WAK and WAKL proteins have WAK domains and protein kinase domains, while WAKs have additional EGF domains compared to WAKLs [7]. Moreover, WAK proteins must have the transmembrane and signal peptide domain [7]. In *J. regia* and its wild related species *J. mandshurica*, the WAK domains were generally located at the N-terminal of WAK/WAKL proteins, the kinase domains were generally located at the C-terminal and the EGF domains of WAKs were usually located near the transmembrane domains (Figure 2b). Our analysis also found that most of the WAK/WAKL members in two selected species had a signal peptide at their N-terminal and some transmembrane domains (Figure 2b), both of which might be related to the function of WAK/WAKL proteins. Sequence alignment and sequence logo analysis of the domain revealed that the cytoplasmic protein kinase domain was more conserved than the extracellular WAK domain (Figure S1). The above analysis of protein domains showed that WAK/WAKL proteins were relatively conserved. To further explore whether *WAK/WAKL* genes were also conserved at the level of gene structure, we performed gene structure analysis (Figure 2b; Table S2).

We found that the *WAK/WAKL* gene structure of *J. regia* and *J. mandshurica* was highly divergent. Previous studies showed that WAK genes in Arabidopsis and cotton usually have 3 exons and 2 introns [7]. However, only 4 out of 11 *JrWAKs* and 1 out of 5 *JmWAKs* contained three exons. The number of exons in *JrWAKs* ranged from 2 to 5 with an average of 4, and the number of exons in *JmWAKs* ranged from 3 to 8 with an average of 5, indicating that the *WAK* gene structure was more variable and had more introns in *J. mandshurica* than in *J. regia*. The number of exons in *JrWAKL* was relatively conservative, ranging from 2 to 5, while the number of exons in *JmWAKL* varies greatly, ranging from 3 to 11 with an average of 6. It was noteworthy that several *WAK/WAKL* members have very long introns, especially *JrWAK3*.

### 3.4. Chromosomal Distribution and Duplication Mode Analysis of the WAK/WAKL Gene Family in J. regia and Its Wild Related Species J. mandshurica 

**Figure 3:**
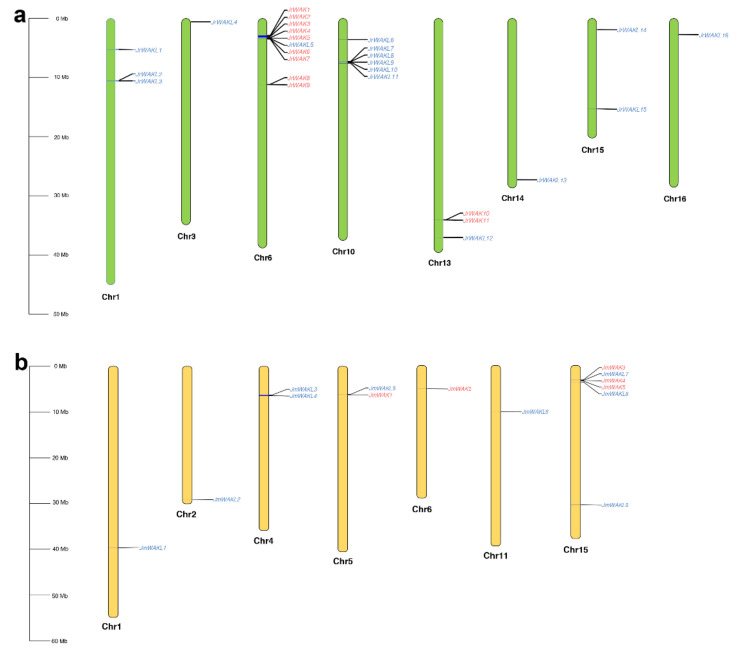
Chromosomal distribution of *WAK/WAKL* genes. (**a**) Chromosomal distribution of *WAK/WAKL* genes in *J. regia*; (**b**) Chromosomal distribution of *WAK/WAKL* genes in *J. mandshurica*. Chromosome numbers were shown below each chromosome. Red, *WAK* genes; blue, *WAKL* genes. Green, chromosomes of *J. regia*; yellow, chromosomes of *J. mandshurica*.

The *JrWAK/WAKL* genes (*WAK/WAKL* genes in *J. regia*) and the *JmWAK/WAKL* genes (*WAK/WAKL* genes in *J. mandshurica*) were located on 8 and 7 chromosomes, respectively (Figure 3). We found that many WAK/WAKLs have tandem duplicated distribution phenomenon.

Based on previous studies, we know that the two *Juglans* species we selected are both diploid, and the number of protein-coding genes is 28,815 and 30,218, respectively [22,23]. However, the number of *WAK/WAKL* genes in *J. regia* (11 + 16 = 27) was nearly twice that in *J. mandshurica* (5 + 9 = 14) in this study. It was evident that the *WAK/WAKL* gene family in *J. regia* was significantly expanded compared to its wild relatives. Therefore, we wanted to explore what types of replications the *JrWAK/WAKL* gene family primarily expands through.

A total of 19 out of 27 *WAK/WAKL* genes in *J. regia* (70.37%) and 9 out of 14 *WAK/WAKL* genes in *J. mandshurica* (64.29%) were tandem repeated genes (Figure 3), which indicated that most of the *WAK/WAKL* genes were tandem duplicated copies in both two *Juglans* species. We also found two *JrWAK/WAKL* tandem repeat clusters on chromosomes 6 and 10 of *J. regia*, respectively. Our previous study found that *J. regia* and *J. mandshurica* had a high degree of collinearity, and homologous chromosomes of JrChr6 and JrChr10 were JmChr15 and JmChr4, respectively [23]. Further analysis showed that 9 *WAK* tandem repeat genes were located on JrChr6 and only 3 *WAK* tandem repeat genes on JmChr15, and 5 WAKL tandem repeat genes were located on JrChr10 and only 2 WAKL tandem repeat genes on JmChr4. Therefore, we preliminarily concluded that tandem duplication (TD) plays an important role in the expansion of the *WAK/WAKL* gene family in *J. regia*.

To further support our hypothesis, we performed a duplication mode analysis (Table 2). There were four modes of duplication events, and the number of TD occurred most frequently (70.37%) in the *WAK/WAKL* gene family in *J. regia*, indicating that TD was indeed the main reason for the expansion of this gene family.

### 3.5. Collinearity and Selective Pressure Analysis of the WAK/WAKL Members in J. regia and Its Wild Related Species J. mandshurica

**Figure 4:**
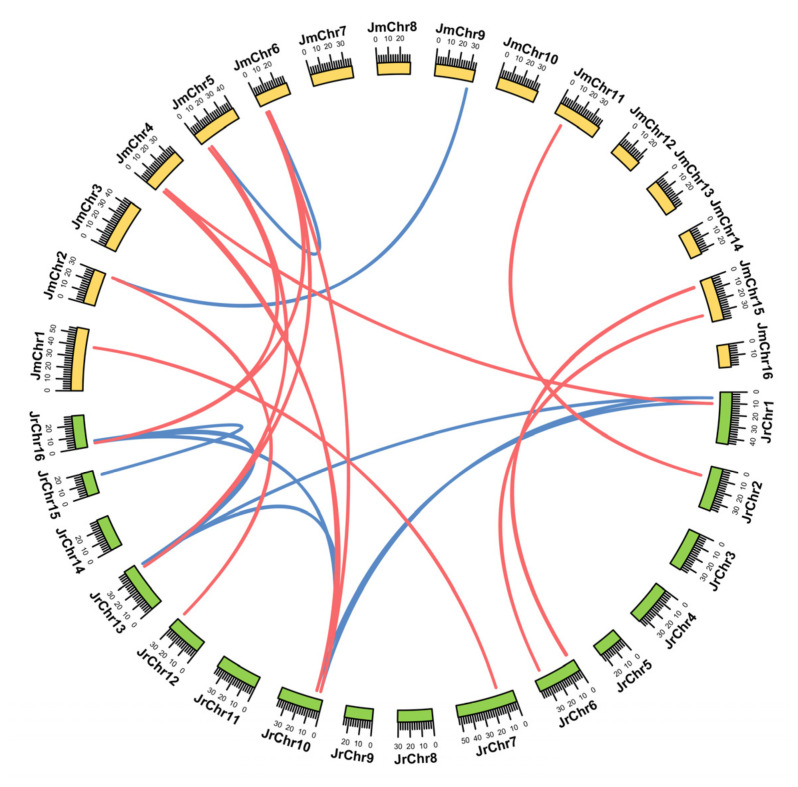
Genome-wide synteny analysis for *WAK/WAKL* genes among *J. regia* and *J. mandshurica*. The orthologous and paralogous *WAK/WAKL* genes were mapped onto the chromosomes and linked by each other. Red lines indicate orthologous gene pairs, blue lines indicate paralogous gene pairs.

Collinearity analysis revealed that there were 22 and 2 *WAK/WAKL* paralogous gene pairs in *J. regia* and *J. mandshurica*, respectively, and 34 *WAK/WAKL* orthologous gene pairs between two *Juglans* species (Figure 4). Clearly, the number of orthologous gene pairs identified was greater than the number of paralogous gene pairs, suggesting a high level of collinearity in *WAK/WAKL* members between *J. regia* and its wild related species *J. mandshurica*, and that most members of the *WAK/WAKL* family might have existed in the ancestors of both *Juglans* species rather than being formed separately after their differentiation. To further explore the selection pressure on these homologous gene pairs, we calculated the Ka/Ks values of these gene pairs (Table S3). The Ka/Ks ratio of most *WAK/WAKL* orthologous pairs was less than 1, indicating that these gene pairs were subjected to purification selection during evolution, and which might limit the functional differentiation of *WAK/WAKL* genes after the differentiation of two *Juglans* species. The Ka/Ks ratio of two homologous gene pairs exceeded 1, indicating that these gene pairs underwent positive selection and might evolve at a relatively fast rate.

### 3.6. Cis-Acting Elements Analysis of Promoters of WAK/WAKL Genes in J. regia and Its Wild Related Species J. mandshurica

**Figure 5:**
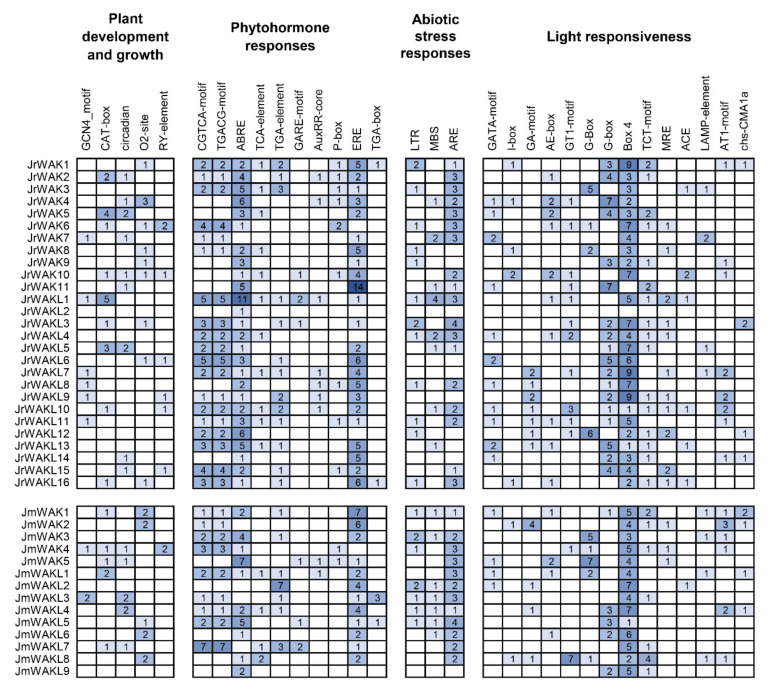
*Cis*-acting elements in promoter regions of *WAK/WAKL* genes in two *Juglans* species. On the basis of functional annotations, the *cis*-acting elements were categorized into four major classes: plant development and growth, phytohormone responses, abiotic stress responses and light responsiveness *cis*-acting elements. The number in the colored box represented the number of *cis*-acting elements.

To investigate the potential function of *WAK/WAKL* genes in *J. regia* and *J. mandshurica*, we analyzed *cis*-acting elements in their upstream promoter regions. We analyzed four types of *cis*-acting elements, including plant development and growth, phytohormone responses, abiotic stress responses and light responsiveness (Figure 5). The promoter regions of most *WAK/WAKL* members contain *cis*-acting elements related to plant growth and development, suggesting that they might play an important role in the growth and development of *J. regia* and its wild related species *J. mandshurica*. In addition, all the *WAK/WAKL* members identified in *J. regia* and *J. mandshurica* contain multiple *cis*-acting elements related to response, suggesting that they might work in plant cell signal transduction. We found that promoters of *JrWAK/WAKL* contained more response elements than that of *JmWAK/WAKL* (Figure 5), suggesting that *WAK/WAKL* in *J. regia* might participate in more complex signal transduction pathways than its wild related species *J. mandshurica*. Three plant hormone response elements were identified in large numbers, namely CGTCA and TGACG motif (associated with Methyl Jasmonate hormone response), and ABRE (associated with Abscisic Acid hormone response), suggesting that *WAK/WAKL* genes in *J. regia* and *J. mandshurica* may be involved in the response of these two hormones.

### 3.7. Expression Profile Analysis of the WAK/WAKL Genes in J. regia and Its Wild Related Species J. mandshurica

**Figure 6:**
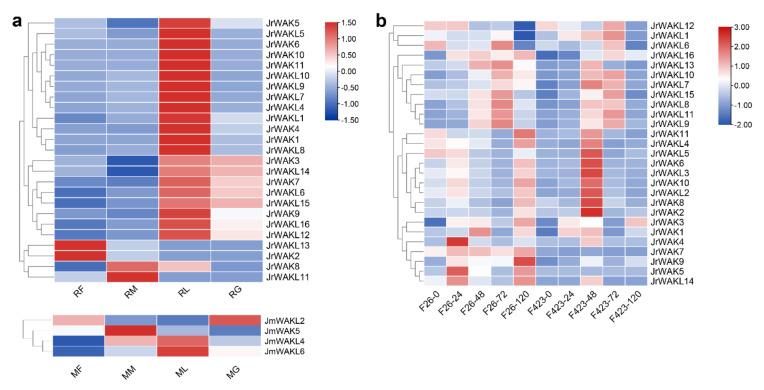
Expression patterns of *WAK/WAKLs* in *J. regia* and *J. mandshurica*. (**a**) Expression patterns of identified *WAK/WAKLs* in female flowers, male flowers, leaves and green pericarp of two *Juglans* species; RF, female flowers of *J. regia*; RM, male flowers of *J. regia*; RL, leaves of *J. regia*; RG, green pericarp of *J. regia*; MF, female flowers of *J. mandshurica*; MM, male flowers of *J. mandshurica*; ML, leaves of *J. mandshurica*; MG, green pericarp of *J. mandshurica*; (**b**) Expression patterns of identified *WAK/WAKL* in *J. regia* under biotic stress. F26 indicated anthracnose-resistant varieties, F423 indicated anthracnose-susceptible varieties. The number after ‘-’ represented the time after infection (unit: hour). The colored scale reflects gene expression levels.

To investigate tissue-specific *WAK/WAKL* expression patterns in *J. regia* and its wildrelated species *J. mandshurica*, transcriptomic data from female flowers, male flowers, leaves and green pericarp of both species were analyzed (Figure 6a; Table S4). We found only 2 *JrWAK/WAKL* genes (7.41%) were not expressed in the selected organs of *J. regia*, while the number was as high as 10 (71.43%) in *J. mandshurica*. This suggested that most *JrWAK/WAKLs* worked in the selected organs, while most *JmWAK/WAKLs* might function in other organs or at other development stages of selected organs. Most *JrWAK/WAKL* genes (21 out of 25) were predominantly expressed in leaves, suggesting that these genes play an important role in the leaf development of *J. regia*. *JrWAK2* and *JrWAKL12* highly expressed in female flowers of *J. regia* and also have similar expression patterns, suggesting that they might play a joint role in female flower development of *J. regia.*

To further investigate the roles of *JrWAK/WAKLs* in the stress response of *J. regia*, we analyzed the gene expression patterns in different varieties of walnuts under biotic stress. Overall, *WAK/WAKL* gene expression levels were higher in anthracnose-resistant varieties (F26 in Figure 6b) than in anthracnose-susceptible varieties (F423) after infection, suggesting that *JrWAK/WAKL* might play roles in walnut resistance to anthracnose. We found that the expression pattern of a cluster of genes (*JrWAKL7-11*, *JrWAKL13*, *JrWAKL15* and *JrWAKL16*) was similar in the two varieties of walnuts. The expression of these genes was induced by the pathogen infection, and increased with the prolongation of infection time within 72 h after infection. In addition, we found that some genes (such as *JrWAK7* and *JrWAK9*) were highly expressed only in the anthracnose-resistant varieties, and the expression levels of them increased with the prolongation of infection time, suggesting that they might be closely related to walnut resistance to anthracnose.

## 4. Discussion

### 4.1. Characteristics of WAK/WAKL Genes in J. regia and J. mandshurica

Both *J. regia* and its wild relative, *J. mandshurica*, are economically important tree species [21]. With the completion of genome sequencing in recent years, it is possible to carry out comparative studies on key gene families in these two species [22,23]. *WAK/WAKL* is a type of RLKs in plants. These proteins can transmit signals received on the cell wall into the cell and thus play an essential role in signal transduction between the cell wall and the cytoplasm [33]. Functional studies of WAK/WAKL in some plants showed that these proteins were mainly involved in plant responses to biological stress [10,11,18]. In addition, our previous study found that the *WAK* gene family in *J. mandshurica* contracted significantly and evolved rapidly compared with *J. regia*, suggesting that this gene family might play an important role in the evolutionary divergence between these two species [23]. Here, we conducted a comparative study on the characteristics of the *WAK/WAKL* gene family in *J. regia* and *J. mandshurica*. The phylogeny, gene structure and expression profiles of the *WAK/WAKL* gene family in *J. regia* and *J. mandshurica* will provide useful information for further research into the gene evolution and candidate genes for studies on *WAK/WAKL* gene function in these two walnut species and *Juglans*.

In this study, a total of 27 and 14 *WAK/WAKL* genes were identified in *J. regia* and *J. mandshurica*, respectively (Figure 1). Phylogenetic analysis shows that these members can be divided into five clades (Figure 1). Previous phylogenetic studies on WAK/WAKL proteins in cotton [7], tomato [34], barley [25] and other plants have found that these proteins tend to form species-specific clades. The most WAK/WAKL proteins in Arabidopsis clustered in the same group compared with other species [7,34,35]. The phylogenetic relationships of five species (Arabidopsis, *G. hirsutum*, *Q. robur*, *J. regia* and *J. mandshurica*) also showed that the WAK/WAKL proteins clustered into species-specific clades, especially Arabidopsis and *G. hirsutum* (Figure 1). These results suggest that WAK/WAKLs might have evolved independently after the differentiation of monocotyledons and dicotyledons speciation. The WAK/WAKLs have a high degree of intra-species conservation and inter-species diversity during the evolution process [3,7]. *WAK/WAKL* members of *J. regia* and *J. mandshurica* have uneven distribution on chromosomes; however, some clusters of *WAK/WAKL* genes had high similarity and collinearity in two walnut species (Figure 1 and Figure 4). This cluster distribution phenomenon has also been observed in cotton, which might prevent the loss of key functions of *WAK/WAKL* in the process of evolution [7].

### 4.2. The Tandem Duplication of the WAK/WAKL Gene Family in J. regia and J. mandshurica

*J. regia* and its wild relative species *J. mandshurica* belong to the same genus, and their genome size and the number of genes is similar [22,23]. However, in this study, we found that the number of *WAK/WAKL* gene family members in *J. regia* was nearly twice that in *J. mandshurica* (Table 1; Figure 1). In other words, the *WAK/WAKL* gene family was significantly expanded in *J. regia* compared to *J. mandshurica*. Polyploidy was an important mechanism of gene family expansion, and tandem duplication (TD) was the common mechanism of the increase in gene copy number [35]. In this study, we found that TD played an important role in the expansion of the *WAK/WAKL* gene family in *J. regia*. In cotton, the TD and segmental duplication may also be responsible for the expansion of the *WAK/WAKL* gene family [7]. Considering that cotton is a polyploid species, segmental duplication (WGD) is often one of the main causes of their gene family expansion [7]. Therefore, it is speculated that TD might be one of the common causes of *WAK/WAKL* gene family expansion in different species. TD was an important single-gene duplication mode, and tandem duplicated genes were closely contiguous on the same chromosome, which might occur through unequal hybridization [36]. A study comparing the RLK family in Arabidopsis and rice found that this family expanded through polyploidy and TD in Arabidopsis, while in rice, this family expanded mainly through TD [37]. They also found that most of the RLKs involved in the defense/resistance response were located in tandem clusters, leading to the hypothesis that genes in the RLK family evolved into a specific response to stress in rice [37]. Interestingly, the potential biotic stress response genes *JrWAK7* and *JrWAK9,* identified by expression profile analysis, were both located in a tandem cluster in this study, suggesting that the *WAK/WAKL* gene family in walnuts might also be consistent with this hypothesis.

Duplicate gene pairs have several different fates, such as neofunctionalization and subfunctionalization [38,39]. The neofunctionalized gene pairs undergo positive selection (Ka/Ks value greater than 1), while subfunctionalized gene pairs undergo purified selection (Ka/Ks value less than 1) [38,39]. In this study, most of the *WAK/WAKL* homologous pairs underwent purified selection, while a few underwent positive selection, suggesting that the evolutionary fate of most *WAK/WAKL* members might be subfunctionalization in *J. regia* and *J. mandshurica*.

### 4.3. The Conserved Domain, Gene Structure, and cis-Acting Elements of the WAK/WAKL Genes in J. regia and J. mandshurica

Typical WAK proteins contain extracellular WAK domain, intracellular protein kinase domain, EGF domain(s), transmembrane domains and signal peptides, while WAKL proteins contain WAK and kinase domains but do not contain the EGF domain [7]. We found that the consistency and conservation of WAK/WAKL proteins in the cytoplasmic region of *J. regia* and *J. mandshurica* was higher than that in the extracellular region (Figure S1), which is also found in Arabidopsis and cotton [7,40]. The similar pattern of WAK/WAKL proteins in different species suggests that its beneficial for WAK/WAKLs to recognize a variety of extracellular ligands or perceive a variety of environmental signals, which is further demonstrating the importance of this family for plant growth and development [7,40]. Although the identified WAK/WAKL protein domains were conserved, their gene structure was different in *J. regia* and *J. mandshurica* (Figure 2), which was quite different from that of Arabidopsis [12]. The *WAK/WAKL* genes were structurally conserved in Arabidopsis (with 3 exons and 2 introns), and the location or length of these 2 introns were also highly conserved [12]. In our study, we found that the number of exons in many *WAK/WAKLs* of *J. regia* and *J. mandshurica* were greater than 3, and several members had very long introns in *J. mandshurica*, such as *JmWAK14*, *JmWAKL3*, *JmWAKL9* and *JmWAKL7* (Figure 2c). This phenomenon was not observed in *WAK/WAKL* genes of other species, so it might be unique to *WAK/WAKLs* of *J. regia* and *J. mandshurica*. Increases in intron number and length for genes might be more beneficial than harmful to plants [41,42]. As the non-coding region, introns could protect genes from mutation interference, so that gene function can be well preserved [41,42]. Importantly, the increase of introns could lead to the increase of protein diversity through alternative splicing, and thereby helping genes play multiple roles in plants [43]. In addition, introns could be involved in the regulation of gene expression by encoding a variety of regulatory factors, and thus might be crucial for the regulation of gene expression in plants [44].

*Cis*-acting elements are involved in the regulation of gene expression, so we analyzed *cis*-acting elements in the identified *WAK/WAKL* promoter regions (Figure 5). In this study, several *cis*-acting elements responding to Methyl Jasmonate (MeJA) and Abscisic Acid (ABA) were identified in the upstream *WAK/WAKL* promoter region (Figure 5), suggesting that WAK/WAKL proteins might be involved in plant responses to MeJA and ABA regulations. Plant hormones are often involved in the response process of plants to stress and are crucial for plants to adapt to adverse environmental conditions [45,46]. Therefore, this result suggests that *WAK/WAKL* might play an important role in stress response of plants. In addition, we detected some *cis*-acting elements associated with abiotic stress response, suggesting that *WAK/WAKLs* might be involved in environmental stimulus response, further supporting their important roles in stress responses. Most *JrWAK/WAKL* genes (21 out of 25) expressed highly in leaves compared to female flowers, male flowers and fruits, however, *JrWAK2* and *JrWAKL12* highly expressed in *J. regia* female flowers, while *JrWAK8* and *JrWAKL11* highly expressed in male flowers (Figure 6a). These results indicated that *JrWAK/WAKL* genes play specific roles in different tissues and organs in *J. regia.* The four *WAK/WAKL* genes *JmWAKL12*, *JmWAK5*, *JmWAKL4* and *JmWAKL6* were expressed highly specific in *J. mandshurica* flowers, leaves and fruits, which are also valuable candidate genes for studies on the development of *J. mandshurica* [15,16]. In addition, the analysis of *JrWAK/WAKL* gene expression patterns after disease treatment of fruits among five development stages also suggested that some *WAK/WAKL* genes might play important roles in the biotic stress response of *J. regia and J. mandshurica* (Figure 6) [10,11,17,18].

## Figures and Tables

**Table 1:** The predicated protein information of *WAK/WAKL* in *J. regia* and *J. mandshurica*.

Gene Name	No. of Amino Acids	Mol. Wt (kDa)	Isoelectric Point (pI)	Instability Index (II)	Aliphatic Index	Grand Average of Hydropathicity (GRAVY)	Subcellular Localizationa
*JrWAK1*	690	77,758.55	5.97	40.56	80.94	−0.31	plas
*JrWAK2*	839	91,544.55	6.36	33.11	82.81	−0.33	plas
*JrWAK3*	778	85,990.06	6.36	37.49	92.57	−0.19	vacu
*JrWAK4*	836	92,128.95	6.67	35.95	83.83	−0.29	plas
*JrWAK5*	778	85,292.05	6.25	31.33	87.90	−0.21	vacu
*JrWAK6*	768	85,036.38	6.96	37.39	91.76	−0.19	plas
*JrWAK7*	716	79,111.38	8.12	34.46	89.15	−0.26	vacu
*JrWAK8*	713	79,985.06	5.93	36.64	83.52	−0.22	vacu
*JrWAK9*	713	79,776.70	5.64	33.72	82.85	−0.24	vacu
*JrWAK10*	763	84,326.97	5.43	36.41	84.84	−0.20	plas
*JrWAK11*	707	78,434.90	8.48	42.21	87.96	−0.21	plas
*JrWAKL1*	673	74,644.43	5.27	43.53	87.74	−0.16	extr
*JrWAKL2*	708	79,775.49	7.86	38.86	84.66	−0.23	chlo
*JrWAKL3*	739	83,259.24	7.71	36.16	84.79	−0.26	extr
*JrWAKL4*	644	70,964.82	8.83	37.45	92.34	−0.13	vacu
*JrWAKL5*	827	91,687.81	7.63	36.59	90.50	−0.16	plas
*JrWAKL6*	694	76,326.10	5.56	36.81	83.16	−0.18	plas
*JrWAKL7*	707	78,717.69	6.33	32.77	86.53	−0.14	plas
*JrWAKL8*	723	81,305.71	6.01	33.79	81.29	−0.23	plas
*JrWAKL9*	513	57,573.86	7.82	27.57	86.80	−0.08	chlo
*JrWAKL10*	705	79,273.50	6.54	28.82	85.97	−0.18	plas
*JrWAKL11*	603	67,641.00	7.76	31.67	82.12	−0.35	nucl
*JrWAKL12*	681	76,621.84	6.37	48.65	83.85	−0.33	plas
*JrWAKL13*	678	75,503.57	5.91	43.32	82.05	−0.24	chlo
*JrWAKL14*	787	87,360.88	6.51	33.75	83.60	−0.23	plas
*JrWAKL15*	669	74,597.36	5.94	39.87	81.46	−0.22	vacu
*JrWAKL16*	670	74,773.49	7.78	46.52	82.19	−0.30	plas
*JmWAK1*	758	84,080.04	6.35	35.01	84.37	−0.21	plas
*JmWAK2*	749	83,946.50	5.86	35.86	82.26	−0.26	vacu
*JmWAK3*	1396	155,950.00	7.06	38.40	87.49	−0.25	plas
*JmWAK4*	763	84,642.55	7.88	40.86	86.09	−0.27	plas
*JmWAK5*	779	85,485.49	6.56	35.61	88.19	−0.22	plas
*JmWAKL1*	629	69,698.84	6.64	51.86	94.10	0.01	plas
*JmWAKL2*	632	69,194.91	6.20	36.32	87.66	−0.16	plas
*JmWAKL3*	1182	133,611.68	6.61	32.72	88.15	−0.22	plas
*JmWAKL4*	881	99,728.80	6.22	35.90	85.27	−0.23	chlo
*JmWAKL5*	706	78,535.72	4.86	36.53	83.81	−0.21	plas
*JmWAKL6*	682	78,128.27	5.79	52.94	83.05	−0.22	plas
*JmWAKL7*	880	98,454.69	8.40	39.81	90.69	−0.23	plas
*JmWAKL8*	662	73,877.35	7.11	42.40	88.34	−0.30	plas
*JmWAKL9*	959	106,500.53	5.56	40.59	95.62	−0.14	plas

Note: plas, plasma membrane; vacu, vacuole; extr, extracellular; chlo, chloroplast; nucl, nucleus.

**Table 2:** The four duplicated types of *WAK/WAKL* genes in *J. regia* and *J. mandshurica*.

Gene Name	Whole Genome Duplication (WGD)	Tandem Duplication (TD)	Dispersed Duplication (DSD)	Proximal Duplication (PD)
*JrWAK1*				√
*JrWAK2*		√		
*JrWAK3*			√	
*JrWAK4*		√		
*JrWAK5*		√		
*JrWAK6*		√		
*JrWAK7*		√		
*JrWAK8*		√		
*JrWAK9*		√		
*JrWAK10*	√	√		
*JrWAK11*		√		
*JrWAKL1*				
*JrWAKL2*		√		
*JrWAKL3*		√		
*JrWAKL4*			√	
*JrWAKL5*		√		
*JrWAKL6*	√	√		
*JrWAKL7*		√		
*JrWAKL8*		√		
*JrWAKL9*		√		
*JrWAKL10*		√		
*JrWAKL11*				√
*JrWAKL12*	√			
*JrWAKL13*		√		
*JrWAKL14*		√		
*JrWAKL15*				
*JrWAKL16*	√			
*JmWAK1*		√		
*JmWAK2*	√			
*JmWAK3*		√		
*JmWAK4*		√		
*JmWAK5*		√		
*JmWAKL1*			√	
*JmWAKL2*	√			
*JmWAKL3*				√
*JmWAKL4*				√
*JmWAKL5*				√
*JmWAKL6*			√	
*JmWAKL7*		√		
*JmWAKL8*		√		
*JmWAKL9*		√		

## Data Availability

The raw data were downloaded from the SRA database under accession number (GSE147083).

## Supplementary Materials

The following are available online at https://www.mdpi.com/article/10.3390/genes13010134/s1, Figure S1: Sequence logos of the protein domains. (a) Sequence logos of the extracellular WAK domain; (b) Sequence logos of the cytoplasmic protein kinase domain, Table S1: The protein sequence of all *WAK/WAKL* genes of two *Juglans* species, Table S2: Number of exons of *WAK/WAKL* genes in *J. regia* and *J. mandshurica*, Table S3: Estimated Ka/Ks ratios of homologous *WAK/WAKL* gene pairs in *J. regia* and *J. mandshurica*, Table S4: The FPKM values of all *WAK/WAKL* genes of two *Juglans* species.
