# Supplementary material for: Identification and Characterization of Wall-Associated Kinase (WAK) and WAK-like (WAKL) Gene Family in Juglans regia and Its Wild Related Species Juglans mandshurica
Source: Genes (Basel). 2022 Jan 12;13(1):134. doi: 10.3390/genes13010134 (PMC8775259; doi:10.3390/genes13010134)
Supplement: Supplementary file 1 [file genes-13-00134-s001.zip › Table S2.pdf]

Table S2 Number of exon of *WAK/WAKL* genes in *Juglans regia* and *J. mandshurica*.

Gene name	Number of exons
JrWAK1	2
JrWAK2	4
JrWAK3	4
JrWAK4	3
JrWAK5	5
JrWAK6	5
JrWAK7	5
JrWAK8	3
JrWAK9	4
JrWAK10	3
JrWAK11	3
JrWAKL1	4
JrWAKL2	3
JrWAKL3	3
JrWAKL4	2
JrWAKL5	5
JrWAKL6	4
JrWAKL7	2
JrWAKL8	3

JrWAKL9	2
JrWAKL10	3
JrWAKL11	3
JrWAKL12	4
JrWAKL13	2
JrWAKL14	3
JrWAKL15	4
JrWAKL16	4
JmWAK1	4
JmWAK2	3
JmWAK3	8
JmWAK4	4
JmWAK5	4
JmWAKL1	3
JmWAKL2	3
JmWAKL3	11
JmWAKL4	8
JmWAKL5	5
JmWAKL6	5
JmWAKL7	5
JmWAKL8	6
JmWAKL9	7
