# Supplementary material for: Identification and Characterization of Wall-Associated Kinase (WAK) and WAK-like (WAKL) Gene Family in Juglans regia and Its Wild Related Species Juglans mandshurica
Source: Genes (Basel). 2022 Jan 12;13(1):134. doi: 10.3390/genes13010134 (PMC8775259; doi:10.3390/genes13010134)
Supplement: Supplementary file 1 [file genes-13-00134-s001.zip › Table S3.pdf]

Table S3 Estimated Ka/Ks ratios of duplicated *WAK/WAKL* gene pairs in *Juglans regia and J. mandshurica.*

Homologous gene pairs			Ks	Ka	Ka/Ks
Jma000010712	vs.	LOC109020209	0.192936	0.386669	0.498969
Jma000010718	vs.	LOC108981954	0.244193	0.764108	0.319579
Jma000027379	vs.	LOC108991622	0.079230	0.101024	0.784267
Jma000010022	vs.	LOC109012425	0.471259	2.678511	0.175941
Jma000027397	vs.	LOC109012425	0.392332	0.863728	0.454231
Jma000028526	vs.	LOC109012490	0.396963	0.812479	0.488582
Jma000010022	vs.	LOC109012490	0.274119	0.499994	0.548244
Jma000012097	vs.	LOC109010810	0.375219	1.855395	0.202231
Jma000010725	vs.	LOC109014610	0.309044	0.476389	0.648721
Jma000028562	vs.	LOC108981029	0.026250	0.063764	0.411665
Jma000027379	vs.	LOC109014992	0.079230	0.101024	0.784267
Jma000027379	vs.	LOC108996119	0.313340	0.848647	0.369223
Jma000010618	vs.	LOC108980353	0.167197	0.277879	0.601688
Jma000021159	vs.	LOC118347739	1.556464	1.406164	1.106887

Jma000010170	vs.	LOC108988286	-	2.527744	-
Jma000001165	vs.	LOC108983353	0.008411	0.033246	0.252984
LOC109010823	vs.	LOC108997248	0.254942	0.727140	0.350609
LOC108996119	vs.	LOC109014992	0.269329	0.736894	0.365492
LOC109012425	vs.	LOC108987648	0.435963	2.479207	0.175848
LOC108988017	vs.	LOC108988017	0.000000	0.000000	-
LOC109012425	vs.	LOC109012490	0.443470	3.244335	0.136690
LOC108987648	vs.	LOC109012490	0.169246	0.483389	0.350123
LOC109010677	vs.	LOC109012490	2.830937	3.088226	0.916687
LOC108988017	vs.	LOC108987648	-	4.008405	-
LOC10901242	vs.	LOC108987648	0.435963	2.479207	0.175848

5					
LOC108987648	vs.	LOC109012490	0.169246	0.483389	0.350123
Jma000028608	vs.	Jma000012097	0.357288	1.902357	0.187813
Jma000001165	vs.	Jma000024408	0.324248	2.451943	0.132241
