# Supplementary material for: Identification and Characterization of Wall-Associated Kinase (WAK) and WAK-like (WAKL) Gene Family in Juglans regia and Its Wild Related Species Juglans mandshurica
Source: Genes (Basel). 2022 Jan 12;13(1):134. doi: 10.3390/genes13010134 (PMC8775259; doi:10.3390/genes13010134)
Supplement: Supplementary file 1 [file Figure S1.pdf › Figure S1.pdf]

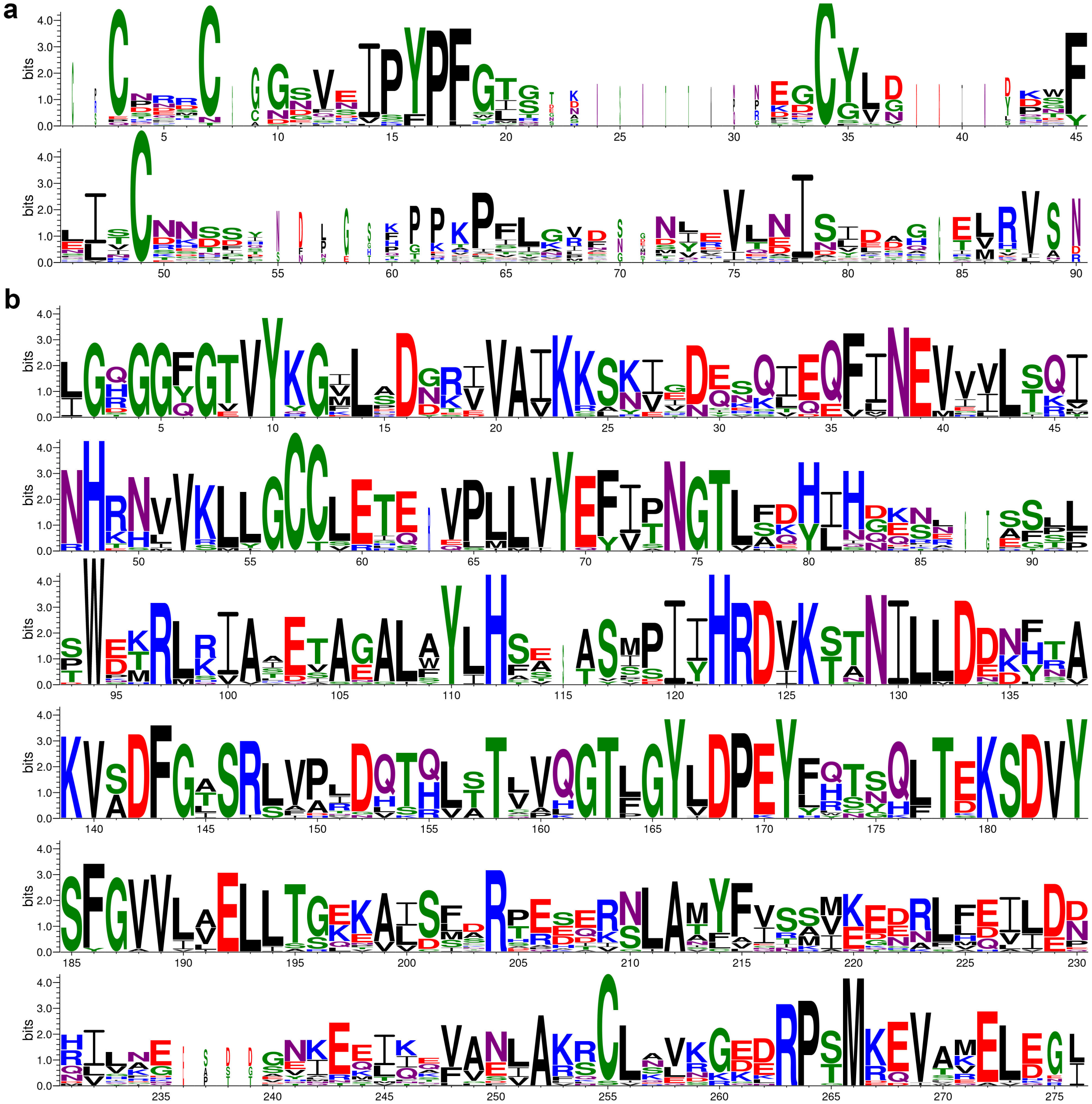
a
b
bits
